# Supplementary material for: Serum albumin and albuminuria predict the progression of chronic kidney disease in patients with newly diagnosed type 2 diabetes: a retrospective study
Source: PeerJ. 2021 Jul 6;9:e11735. doi: 10.7717/peerj.11735 (PMC8269640; doi:10.7717/peerj.11735)
Supplement: Supplemental Information 2 [file peerj-09-11735-s002.docx]

**Code book**

SEX: 1 Male 2 Female

Smoking: 1 Yes 2 No

Drinking: 1 Yes 2 No

Hypertension: 1 Yes 2 No

Coronary heart disease: 1 Yes 2 No

Cerebrovascular diseases: 1 Yes 2 No

Progression of CKD: 1 Yes 2 No

Quartiles(Albuminuria):

>300 4

100-300 3

30-100 2

<30 mg/24 h 1
